# A Characterization of Pediatric Burn Injury Patients Presenting to a Zonal Referral Hospital in Northern Tanzania

**DOI:** 10.1093/jbcr/iraf184

**Published:** 2025-09-30

**Authors:** Kajsa Vlasic, Theresia Mwakyembe, Francis Sakita, Irma Fleming, Giavonni Lewis, Getrude Nkini, Nancy Mmary, Deus Marandu, Nora Fino, Jonah Holiday, Melissa H Watt, Catherine A Staton, Elizabeth M Keating, Blandina M Mmbaga

**Affiliations:** Division of Pediatric Emergency Medicine, Department of Pediatrics, University of Utah, Salt Lake City, UT, United States; Global Emergency Medicine Innovation and Implementation (GEMINI) Research Center, Duke University Medical Center, Durham, NC, United States; Kilimanjaro Christian Medical Centre, Moshi, Tanzania; School of Medicine, KCMC University, Moshi, Tanzania; Kilimanjaro Christian Medical Centre, Moshi, Tanzania; School of Medicine, KCMC University, Moshi, Tanzania; Department of Surgery, University of Utah, Salt Lake City, UT, United States; Department of Surgery, University of Utah, Salt Lake City, UT, United States; Kilimanjaro Christian Medical Centre, Moshi, Tanzania; KCMC-Duke Collaboration, Moshi, Tanzania; Kilimanjaro Christian Medical Centre, Moshi, Tanzania; KCMC-Duke Collaboration, Moshi, Tanzania; Kilimanjaro Christian Medical Centre, Moshi, Tanzania; KCMC-Duke Collaboration, Moshi, Tanzania; Division of Epidemiology, Department of Internal Medicine, University of Utah, Salt Lake City, UT, United States; Arizona State University, Tempe, AZ, United States; Department of Population Health Sciences, University of Utah, Salt Lake City, UT, United States; Global Emergency Medicine Innovation and Implementation (GEMINI) Research Center, Duke University Medical Center, Durham, NC, United States; Duke Global Health Institute, Duke University, Durham, NC, United States; Department of Emergency Medicine, Duke University Medical Center, Durham, NC, United States; Division of Pediatric Emergency Medicine, Department of Pediatrics, University of Utah, Salt Lake City, UT, United States; Global Emergency Medicine Innovation and Implementation (GEMINI) Research Center, Duke University Medical Center, Durham, NC, United States; Global Emergency Medicine Innovation and Implementation (GEMINI) Research Center, Duke University Medical Center, Durham, NC, United States; Kilimanjaro Christian Medical Centre, Moshi, Tanzania; School of Medicine, KCMC University, Moshi, Tanzania; Kilimanjaro Clinical Research Institute, Moshi, Tanzania

**Keywords:** pediatric burn injuries, burn severity, Tanzania, in-hospital mortality, pre-hospital care

## Abstract

Burn injuries disproportionately impact children in low- and middle-income countries (LMICs), with Sub-Saharan Africa bearing the highest burden. While pediatric injury research in LMICs is growing, data on pediatric burn injuries and associated mortality in LMICs remain limited. The objective of this study was to describe a cohort of pediatric burn injury patients from a pediatric injury registry in Northern Tanzania, including the epidemiology, clinical presentation, pre-hospital factors, and clinical outcomes. We conducted a retrospective observational study of burn injury patients from a pediatric injury registry at a tertiary zonal referral hospital in Northern Tanzania. We evaluated patient demographics, emergency department presentation, and inpatient data. Study outcomes included ICU admission, in-hospital mortality, and morbidity at discharge. Differences in statistics were evaluated with analysis of variance (ANOVA)/*t*-test, chi-square tests, or Fisher’s exact tests. We assessed associations with ICU stay, in-hospital mortality, and morbidity by reporting adjusted odds ratios and 95% confidence intervals from multivariable logistic regression models. 133 pediatric burn patients were enrolled between November 2020 and August 2024. Sixty-eight patients were female (51%), and most were aged 5 years or younger (81.9%). Scalds were the most common injury. The in-hospital mortality rate was 22.6%. Burn severity was independently associated with all 3 study outcomes. Pediatric burn mortality was high, with burn severity predicting poor outcomes. This study highlights the urgent need to address gaps in burn injury prevention, community education on timely burn care, and pre-hospital and referral systems for pediatric burn patients in Northern Tanzania.

## INTRODUCTION

The landscape of global pediatric health has shifted in recent decades, with injuries accounting for a growing proportion of morbidity and mortality given advances in infection control, nutrition, and vaccinations.^1^^,^^2^ Studies have shown that injuries, including burns, are a global public health issue.^3^^,^^4^ Specifically, burn injuries disproportionately impact children in low- and middle-income countries (LMICs), with the highest incidence of pediatric burn injury occurring in Sub-Saharan Africa.^5^^,^^6^ For children who survive burn injuries, there is potential for lasting effects on quality of life, particularly in resource-limited areas.^7^^,^^8^

Burn injuries have been studied in East Africa previously; however, the data is still limited.^5^^,^^6^^,^^9^^,^^10^ A literature review of pediatric burn studies from Tanzania highlighted burns primarily impacting young children across the country and most often occurring around cooking fires.^8^ Like data from other LMIC settings, the mortality rate of pediatric burn injuries in East Africa is high, and for those who survive, there are long-lasting effects.^11^ A recent study described the findings of a pediatric injury registry at a tertiary-level hospital in Northern Tanzania with an overall mortality rate of 8.2% for all admitted pediatric injury patients and 43% of those deaths due specifically to burns.^12^ A cross-sectional study of pediatric burn injuries at this same hospital in Northern Tanzania performed 10 years ago showed a predominance of children under the age of 5 years, more than half of the burns due to flame, and a high mortality rate of 26.8%.^13^ Since this study was performed, the healthcare system in Tanzania has changed substantially, prompting the need for an updated and current assessment to understand areas for improved pediatric burn care.

To address this gap, we evaluated a cohort of pediatric burn injury patients enrolled in a prospective pediatric injury registry at a zonal referral hospital with subspecialty burn treatment capability in Northern Tanzania. The objective of this study was to describe the epidemiology, clinical presentation, pre-hospital factors, and hospital outcomes of this group of pediatric burn injury patients and examine the association of clinical and pre-hospital factors with ICU admission, in-hospital mortality, and morbidity at discharge. The results of this study can identify opportunities for intervention to improve mortality and morbidity outcomes among pediatric burn patients in Northern Tanzania and contribute to improved understanding of pediatric burn injuries in similar LMIC settings globally.

## MATERIALS AND METHODS

### Ethical approvals

This study was approved by the Tanzanian National Institute for Medical Research (NIMR/HQ/R.8a/Vol.IX/3475; approved July 20, 2020), Kilimanjaro Christian Medical University College Institutional Review Board (1252; approved October 19, 2020), and the University of Utah Institutional Review Board (IRB_00134560; approved September 1, 2020).

### Study design

This was a retrospective observational study using a prospectively enrolling pediatric injury registry at Kilimanjaro Christian Medical Centre (KCMC) in Moshi, Tanzania. The pediatric injury registry has been actively enrolling since November 2020. Our analysis included a cohort of 133 pediatric burn injury patients under the age of 18 years enrolled in the registry between November 2020 and August 2024.

### Study setting

Tanzania is an East African country with a population of nearly 62 million.^14^ A majority of the national population (60%) is under the age of 24 years.^15^ KCMC is a tertiary zonal referral hospital located in the Kilimanjaro Region of the Northern Zone of Tanzania. The hospital provides specialty care services for pediatric patients, including burn care. KCMC has a catchment area of 12 million individuals, with the Emergency Medical Department (EMD) seeing approximately 1500 pediatric patients annually. Pediatric and adult burn patients are specifically cared for in the EMD, inpatient Burn Ward, and the Surgical Intensive Care Unit (SICU). The Burn Ward has a capacity of 24 beds, and the SICU has a capacity of 12 beds. Pediatric and adult patients are seen in both locations. Inpatient burn care is provided by 2 general surgeons, 10-12 nurses, 1-2 nutritionists, 2 physical and/or occupational therapists, 1 social worker, and the hospital chaplain team. Inpatient psychiatrists are commonly consulted for admitted burn patients as well.

**Table 1:** Pediatric Injury Registry Data Collected During Hospital Stay and at Discharge

**Acute presentation**	Mechanism of burn injury^a^ Vital signs Total body surface area (TBSA) Mode of transportation to hospital Patient demographics
**Hospital-based care**	Treatment Procedures and operations Location of in-hospital care (Burn Ward, SICU) Complications Length of hospital stay
**Outcomes**	Mortality GOS-E Peds^22^ at time of discharge Consciousness Independence in the home Independence outside the home School/work Social and leisure activities Family and friendships Return to normal life

^a^Mechanism of burn injury data was only collected on patients starting in July 2023. Abbreviations: GOS-E Peds, Glasgow Outcome Score-Extended Pediatrics; SICU, Surgical Intensive Care Unit.

### Study population

The KCMC pediatric injury registry includes patients under the age of 18 years who seek care for an injury that occurred within 1 month of arrival to the EMD at KCMC. The registry excludes patients who present with injuries that occurred more than 1 month prior to presentation or those who are presenting for follow-up care. For the purpose of our study, we included only patients who were enrolled in the KCMC pediatric injury registry with burn injuries. None of our patients had missing outcomes data. We defined burn injuries according to the World Health Organization (WHO): “an injury to the skin or other organic tissue primarily caused by heat or due to radiation, radioactivity, electricity, friction or contact with chemicals”.^16^

### Data collection

Registry data was collected by trained Tanzanian research assistants. Data was collected by direct observation of patient care in the EMD. If children were evaluated outside of data collection times, patients were enrolled the following day if the child was admitted. Children discharged from the EMD outside of data collection times were missed from the registry. As this study is part of a standard healthcare quality improvement process, patients and/or caregivers were not consented.^17^^,^^18^ The registry was observational and did not affect patient care provided, thus consent was implied in the consent to treatment. Research assistants followed patients throughout their hospital course after admission and collected inpatient care and discharge information from the Burn Ward and Surgical Intensive Care Unit. Study data was collected and managed using REDCap © electronic data capture tools hosted at Kilimanjaro Clinical Research Institute.^19^^,^^20^ The research assistants observed patient care, assisted with gathering vital signs, and obtained data from the medical records for registry inclusion. Quality control was performed by the pediatric injury registry principal investigator (E.M.K.), who reviewed all patient entries. Research assistants had prior research experience with specific training on registry data collection and participated in weekly meetings with the research team to evaluate data collection and discuss quality challenges that needed to be addressed.

### Measures

Data collected for our pediatric burn injury cohort reflected original pediatric injury registry work performed at the institution.^12^ Research assistants collected patient demographics, acute presentation information from the EMD, inpatient hospital-based care, and clinical outcomes either through direct conversation with patients and family or through chart review (Table 1). Initial vital signs were recorded at EMD arrival. We defined age-specific vital sign abnormalities as seen in Table 2. Burn severity was classified based on total body surface area (TBSA), as this was a variable consistently documented in the patient chart. TBSA data was collected via research assistants directly from chart review documentation by either EMD physicians or physicians on the burn team. Burn injuries were defined as *mild* if the TBSA involvement was less than 10%, *moderate* if the TBSA involvement was 10%-19%, and *severe* if the TBSA involvement was greater than or equal to 20%.^21^

**Table 2:** Age-Specific Vital Sign Abnormalities Collected as Part of the Pediatric Registry Data

**Variable**	**Age**	**Abnormal vital sign**
**Hypotension**	0-28 days	Systolic blood pressure < 60 mm Hg
	1-12 months	Systolic blood pressure < 70 mm Hg
	1-10 years	Systolic blood pressure < (70 + [age in years x 2]) mm Hg
	>10 years	Systolic blood pressure < 90 mm Hg
**Hypoxemia**	All ages	Pulse oxygenation <90%
**Tachycardia**	0-3 months	Heart rate > 205 beats per minute
	3 months-2 years	Heart rate > 190 beats per minute
	2 – 10 years	Heart rate > 140 beats per minute
	> 10 years	Heart rate > 100 beats per minute
**SIRS criteria**	*>* 2 of the following (one of which must be abnormal temperature): - Tachycardia - Tachypnea (or mechanical ventilation) - Temperature abnormality *Laboratory data not available in registry*	

Our primary outcomes of interest were ICU admission, in-hospital mortality, and morbidity at time of discharge. Morbidity was measured using the Glasgow Outcome Score-Extended Pediatrics (GOS-E Peds), which is a previously validated external assessment tool where the researcher determines a patient’s capacity after asking the child’s caregiver a number of questions about their functional status at the time of discharge.^22^ The GOS-E Peds is an 8-item instrument and was initially designed to measure outcomes in pediatric traumatic brain injury patients. Since the tool’s initial development, it has been validated in other pediatric trauma populations and has been considered useful for pediatric populations, as it can be answered by a caregiver on behalf of their child.^23^ The tool includes most domains from the WHO’s International Classification of Functioning, Disability and Health and has been recommended for use in trauma registries to monitor changes in functional outcomes.^23^^,^^24^ For this study, we used a dichotomized score with a score of 1-2 representing *good recovery* and a score of greater than 2 representing *poor recovery* for consistency with previous injury work where the GOS-E Peds score was used as a measure of morbidity.^12^

### Statistical methods

We summarized patient demographics, acute presentation information, inpatient hospital-based care, and outcome variables overall and by ICU admission, morbidity, and in-hospital mortality using descriptive statistics. We summarized continuous data as means with standard deviation and nominal data as frequencies and percentages. We assessed for differences in these statistics by ICU admission, in-hospital mortality, and morbidity using analysis of variance (ANOVA) or chi-squared testing where appropriate. We assessed associations with in-hospital mortality, morbidity, and ICU stay by reporting adjusted odds ratios and 95% confidence intervals from multivariable logistic regression models. Analyses were performed in SAS (version 9.4) © and figures were made in R using ggplot2.

## RESULTS

### Demographics

A total of 133 pediatric burn injury patients were enrolled in the pediatric injury registry between November 2020 and August 2024. Burn patients represented 12.6% of the total pediatric injury registry enrollment during this timeframe (*n* = 1058). A slight majority of patients were female (*n* = 68, 51%) and 81.9% (*n* = 109) were aged 5 years or younger, with a mean age of 3.5 years (SD = 3.4 years). Complete demographic data of our study population is presented in Table 3.

**Table 3:** Demographics, Clinical Characteristics, and Pre-Hospital Characteristics

	**Overall**	**Admission to the ICU**	**Did not go the ICU**	***P***	**In-hospital mortality**	**Discharged alive**	***P***	**Good recovery (GOS-E score 1-2)**	**Poor recovery (GOS-E score 3-8)**	***P***
	(*n* = 133)	(*n* = 30)	(*n* = 103)		(*n* = 30)	(*n* = 103)		(*n* = 80)	(*n* = 53)	
**Demographics**										
**Sex, *N* (%)**				.490			.269			.167
Male	65 (48.9%)	13 (20.0%)	52 (80.0%)		12 (18.5%)	53 (81.5%)		43 (66.2%)	22 (33.8%)	
Female	68 (51.1%)	17 (25.0%)	51 (75.0%)		18 (26.5%)	50 (73.5%)		37 (54.4%)	31 (45.6%)	
**Age, years, mean (SD)**	3.5 (3.4)	4.9 (4.7)	3.1 (2.9)	.012	3.8 (3.2)	3.5 (3.5)	.671	3.4 (3.7)	3.8 (3.0)	.495
**Age group, *N* (%)**				.025^b^			.613^b^			.402^b^
1. Infant (0-1 year)	45 (33.8%)	7 (15.6%)	38 (84.4%)		8 (17.8%)	37 (82.2%)		31 (68.9%)	14 (31.1%)	
2. Toddler (2-3 years)	40 (30.1%)	9 (22.5%)	31 (77.5%)		9 (22.5%)	31 (77.5%)		24 (60.0%)	16 (40.0%)	
3. Preschool (4-5 years)	24 (18.0%)	7 (29.2%)	17 (70.8%)		8 (33.3%)	16 (66.7%)		12 (50.0%)	12 (50.0%)	
4. Child (6-11 years)	17 (12.8%)	2 (11.8%)	15 (88.2%)		3 (17.6%)	14 (82.4%)		8 (47.1%)	9 (52.9%)	
6. Teen (12-17 years)	7 (5.3%)	5 (71.4%)	2 (28.6%)		2 (28.6%)	5 (71.4%)		5 (71.4%)	2 (28.6%)	
**Where patient lives, *N* (%)**				.274			.025			.005
Moshi urban district	47 (35.6%)	7 (14.9%)	40 (85.1%)		5 (10.6%)	42 (89.4%)		36 (76.6%)	11 (23.4%)	
Moshi rural district	30 (22.7%)	9 (30.0%)	21 (70.0%)		11 (36.7%)	19 (63.3%)		12 (40.0%)	18 (60.0%)	
Other (rural surrounding areas)	55 (41.7%)	13 (23.6%)	42 (76.4%)		13 (23.6%)	42 (76.4%)		32 (58.2%)	23 (41.8%)	
**Patient lives with, *N* (%)**				.181			.237			.043
Single parent	25 (18.9%)	7 (28.0%)	18 (72.0%)		8 (32.0%)	17 (68.0%)		10 (40.0%)	15 (60.0%)	
Both parents	94 (71.2%)	17 (18.1%)	77 (81.9%)		17 (18.1%)	77 (81.9%)		63 (67.0%)	31 (33.0%)	
Grandparent/aunt/uncle	13 (9.8%)	5 (38.5%)	8 (61.5%)		4 (30.8%)	9 (69.2%)		7 (53.8%)	6 (46.2%)	
**Number of children living in the home, *N* (%)**				.033^b^			.101^b^			.010^b^
1 (patients)	31 (23.5%)	3 (9.7%)	28 (90.3%)		3 (9.7%)	28 (90.3%)		24 (77.4%)	7 (22.6%)	
2-3	75 (56.8%)	18 (24.0%)	57 (76.0%)		18 (24.0%)	57 (76.0%)		44 (58.7%)	31 (41.3%)	
4-5	19 (14.4%)	7 (36.8%)	12 (63.2%)		6 (31.6%)	13 (68.4%)		9 (47.4%)	10 (52.6%)	
6-7	6 (4.5%)	0 (0.0%)	6 (100.0%)		1 (16.7%)	5 (83.3%)		3 (50.0%)	3 (50.0%)	
>7	1 (0.8%)	1 (100.0%)	0 (0.0%)		1 (100.0%)	0 (0.0%)		0 (0.0%)	1 (100.0%)	
**Clinical characteristics**										
**Burn severity, *N* (%)**				<.0001^b^			<.0001^b^			<.0001^b^
1. Mild burn (<10% BSA)	36 (27.1%)	0 (0.0%)	36 (100.0%)		0 (0.0%)	36 (100.0%)		28 (77.8%)	8 (22.2%)	
2. Moderate burn (10%-19% BSA)	37 (27.8%)	4 (10.8%)	33 (89.2%)		4 (10.8%)	33 (89.2%)		27 (73.0%)	10 (27.0%)	
3. Extensive burn (20%-39% BSA)	32 (24.1%)	4 (12.5%)	28 (87.5%)		7 (21.9%)	25 (78.1%)		18 (56.3%)	14 (43.8%)	
4. Severe burn (≥40% BSA)	28 (21.1%)	22 (78.6%)	6 (21.4%)		19 (67.9%)	9 (32.1%)		7 (25.0%)	21 (75.0%)	
**Temperature, *N* (%)** (4 patients missing data)				.003^b^			.010^b^			.344
Hypothermia (<36 °C)	8 (6.2%)	5 (62.5%)	3 (37.5%)		5 (62.5%)	3 (37.5%)		3 (37.5%)	5 (62.5%)	
Normal (36-38 °C)	107 (82.9%)	23 (21.5%)	84 (78.5%)		22 (20.6%)	85 (79.4%)		65 (60.7%)	42 (39.3%)	
Fever (>38C)	14 (10.9%)	0 (0.0%)	14 (100.0%)		1 (7.1%)	13 (92.9%)		10 (71.4%)	4 (28.6%)	
**Tachypnea, *N* (%)**				.289			1^b^			.990
No	118 (88.7%)	25 (21.2%)	93 (78.8%)		27 (22.9%)	91 (77.1%)		71 (60.2%)	47 (39.8%)	
Yes	15 (11.3%)	5 (33.3%)	10 (66.7%)		3 (20.0%)	12 (80.0%)		9 (60.0%)	6 (40.0%)	
**Hypotension, *N* (%)** (24 patients missing data)				.0021			.002			.0221
No	105 (96.3%)	21 (20.0%)	84 (80.0%)		20 (19.0%)	85 (81.0%)		66 (62.9%)	39 (37.1%)	
Yes	4 (3.7%)	4 (100.0%)	0 (0.0%)		4 (100.0%)	0 (0.0%)		0 (0.0%)	4 (100.0%)	
**Hypoxemia, *N* (%)** (2 patients missing data)				.016			.004^b^			.029^b^
No	122 (93.1%)	25 (20.5%)	97 (79.5%)		23 (18.9%)	99 (81.1%)		77 (63.1%)	45 (36.9%)	
Yes	9 (6.9%)	5 (55.6%)	4 (44.4%)		6 (66.7%)	3 (33.3%)		2 (22.2%)	7 (77.8%)	
**Tachycardia, *N* (%)**				.686			.686			.567
No	118 (88.7%)	26 (22.0%)	92 (78.0%)		26 (22.0%)	92 (78.0%)		72 (61.0%)	46 (39.0%)	
Yes	15 (11.3%)	4 (26.7%)	11 (73.3%)		4 (26.7%)	11 (73.3%)		8 (53.3%)	7 (46.7%)	
**Required intubation in the ED, *N* (%)**				<.0001^b^			<.0001^b^			<.0001^b^
No	121 (91.0%)	18 (14.9%)	103 (85.1%)		18 (14.9%)	103 (85.1%)		80 (66.1%)	41 (33.9%)	
Yes	12 (9.0%)	12 (100.0%)	0 (0.0%)		12 (100.0%)	0 (0.0%)		0 (0.0%)	12 (100.0%)	
**Patients meeting SIRS criteria** ^a^ **upon ED arrival, *N* (%)**				.116^b^			.104			.156^b^
No	124 (93.2%)	26 (21.0%)	98 (79.0%)		26 (21.0%)	98 (79.0%)		77 (62.1%)	47 (37.9%)	
Yes	9 (6.8%)	4 (44.4%)	5 (55.6%)		4 (44.4%)	5 (55.6%)		3 (33.3%)	6 (66.7%)	
**Patients meeting SIRS criteria** ^a^ **+ Hypotension upon ED arrival, *N* (%)**				NA^c^			NA^c^			NA^c^
No	132 (99.2%)	29 (22.0%)	103 (78.0%)		29 (22.0%)	103 (78.0%)		80 (60.6%)	52 (39.4%)	
Yes	1 (0.8%)	1 (100.0%)	0 (0.0%)		1 (100.0%)	0 (0.0%)		0 (0.0%)	1 (100.0%)	
**Pre-hospital characteristics**										
**Mechanism of transport to KCMC, *N* (%)**				.007^b^			.029^b^			.093^b^
Ambulance from other hospital	87 (65.4%)	25 (28.7%)	62 (71.3%)		24 (27.6%)	63 (72.4%)		47 (54.0%)	40 (46.0%)	
Private car	27 (20.3%)	2 (7.4%)	25 (92.6%)		3 (11.1%)	24 (88.9%)		19 (70.4%)	8 (29.6%)	
Hired transportation	16 (12.0%)	1 (6.3%)	15 (93.8%)		1 (6.3%)	15 (93.8%)		13 (81.3%)	3 (18.8%)	
Other	3 (2.3%)	2 (66.7%)	1 (33.3%)		2 (66.7%)	1 (33.3%)		1 (33.3%)	2 (66.7%)	
**First health facility treated at, *N* (%)** (33 patients missing data)				.012^b^			.180^b^			.236
KCMC	25 (25.0%)	2 (8.0%)	23 (92.0%)		2 (8.0%)	23 (92.0%)		19 (76.0%)	6 (24.0%)	
Dispensary/health center	25 (25.0%)	2 (8.0%)	23 (92.0%)		6 (24.0%)	19 (76.0%)		15 (60.0%)	10 (40.0%)	
District/regional hospital	50 (50.0%)	16 (32.0%)	34 (68.0%)		13 (26.0%)	37 (74.0%)		28 (56.0%)	22 (44.0%)	
**Time between injury & arrival to KCMC, *N* (%)**										
<8 hours	56 (42.1%)	13 (23.2%)	43 (76.8%)	.705	12 (21.4%)	44 (78.6%)	.349	37 (66.1%)	19 (33.9%)	.363
=8-24 hours	13 (9.8%)	4 (30.8%)	9 (69.2%)		5 (38.5%)	8 (61.5%)		6 (46.2%)	7 (53.8%)	
≥24 hours	64 (48.1%)	13 (20.3%)	51 (79.7%)		13 (20.3%)	51 (79.7%)		37 (57.8%)	27 (42.2%)	

^a^SIRS criteria: *>*2 of the following (1 of which must be abnormal temperature): tachycardia, tachypnea (or mechanical ventilation), temperature abnormality; laboratory data not available in registry. Abbreviations: BSA, body surface area; GOS-E, Glasgow Outcome Score-Extended; KCMC, Kilimanjaro Christian Medical Centre.

*P* values assessed using chi-square test of association (categorical) or analysis of variance/*t*-test (continuous), unless otherwise noted

^b^ *P* value assessed using Fisher’s exact test

^c^Not enough events for valid statistical inference

### Mechanism of burn injury

Cause of burn injury data was collected in 33 patients of the total cohort, as this was a variable that our study team started collecting in the registry in July 2023. The majority of burn injuries were secondary to scald (*n* = 14, 42.4%), followed by open flame (*n* = 8, 24.2%), hot objects (*n* = 8, 24.2%), and chemicals (*n* = 3, 9.2%).

### ICU admission

Thirty patients (22.6%) were admitted to the ICU. ICU admission rates varied significantly by age group and were highest among adolescents aged 12-17 years (71.4%) and lowest in children aged 6-11 years (11.8%). Children from larger households had a higher likelihood of requiring ICU care (*P* = .033).

Clinical factors associated with ICU admission included burn severity, temperature abnormality, hypotension, hypoxemia, and intubation. For every 10% increase in TBSA, the odds of ICU admission more than doubled (OR 2.21, 95% CI, 1.62-3.01, *P* < .0001). Nearly 63% of patients with hypothermia required ICU care compared to 21.5% of patients with normal temperature and none with fever (*P* = .003). Most patients in the cohort (65.4%) were transported to KCMC by ambulance, and patients who arrived via ambulance had a higher likelihood of ICU admission (*P* = .007). Patients first treated at district or regional hospitals were more likely to require ICU care than those seen at KCMC, local dispensaries, or health centers initially (*P* = .012).

### In-hospital mortality

The overall in-hospital mortality rate of our pediatric burn cohort was 22.6% (*n* = 30). Although thirty patients were admitted to the ICU and thirty patients died during hospitalization, as presented in Table 3, these were not the same thirty patients in both groups. Nearly 37% (*n* = 11) of patients from rural areas of Moshi died during hospitalization compared to 10.6% of patients from urban Moshi (*n* = 5) and 23.6% (*n* = 13) of patients from outside of the Moshi area (*P* = .025).

Factors associated with in-hospital mortality were burn severity, temperature abnormality, hypotension, hypoxemia, intubation, mechanism of transport to KCMC, and the first health facility treated at. For every 10% increase in TBSA, patients had 2.58 higher odds of dying during hospitalization (95% CI, 1.79-3.73, *P* < .0001). Patients with hypoxemia had a much higher in-hospital mortality rate than those without (67% vs 19%, *P* = .004). Hypotension and intubation on arrival were strong predictors of poor outcomes, with all 4 hypotensive patients (*n* = 4) and intubated patients (*n* = 12) requiring ICU care and then dying during hospitalization (*P* = .002 and *P* < .0001, respectively). Patients brought by ambulance from another facility had a higher likelihood of in-hospital mortality when compared to patients brought by private car or hired transportation (*P* = .029).

### Morbidity

The GOS-E Peds score (1-2 = good recovery; 3-9 = poor recovery) was used to assess morbidity. The majority of our study cohort (*n* = 80, 60.2%) had GOS-E Peds scores of 1 or 2 at discharge, consistent with good functional recovery. Patients from urban Moshi and further areas of Northern Tanzania were more likely to have better recovery outcomes compared to those from rural Moshi (*P* = .005). In addition, living with both parents and having fewer children in the household were significantly associated with better morbidity outcomes (*P* = .043 and *P* = .010, respectively).

Factors associated with morbidity were burn severity, hypotension, hypoxemia, and intubation. For every 10% increase in TBSA, patients had 1.54 higher odds of poor functional recovery (95% CI, 1.24-1.91, *P* < .0001).

### Impact of burn severity on clinical outcomes

**Figure 1:**
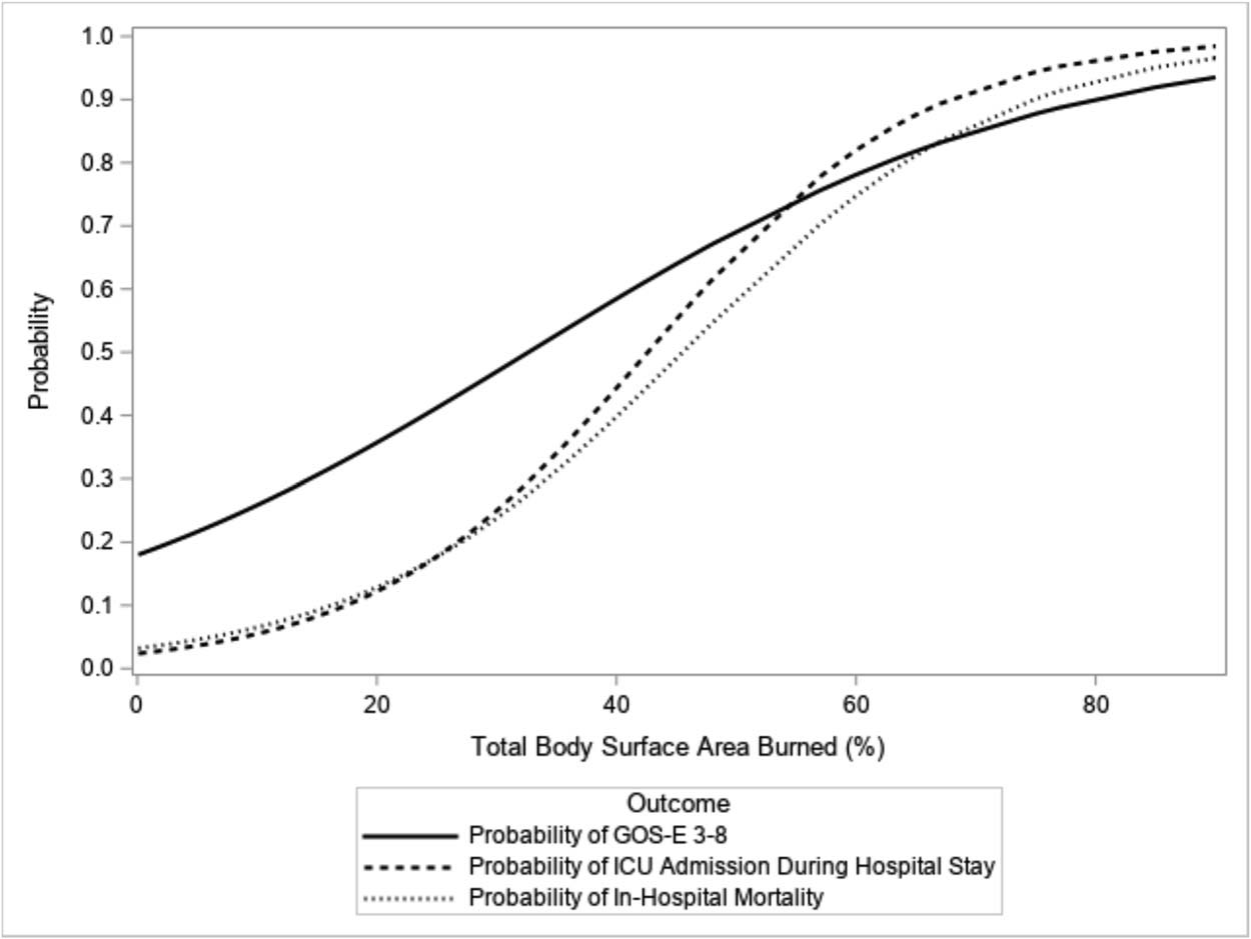
Estimated Probability of Morbidity (GOS-E), ICU Admission, and In-Hospital Mortality by Percent of Total Body Surface Area (TBSA) Burn Injury. Abbreviation: GOS-E, Glasgow Outcome Score-Extended.

Patients with extensive (20%-39%) and severe burns (*>*40% TBSA) made up 24.1% and 21.1% of the study cohort, respectively. Increasing burn severity was independently associated with all 3 outcomes of interest (Table 4). In-hospital mortality was zero for mild burns, 10.8% for moderate burns, 21.9% for extensive burns, and 67.9% for severe burns. For patients in the extensive burn group (20%-39% TBSA), 56.3% of patients had good function at discharge compared to 25% with good function in the severe burn group. A notable inflection point for ICU admission and in-hospital mortality was observed at approximately 25% TBSA on logistic curve modeling (Figure 1). This data underscores that patients presenting with burns between 25% and 40% benefit more from intensive care intervention with the burn team at KCMC. In-hospital mortality rates in patients with TBSA above 40% are strikingly higher than the extensive burn group, likely impacted by limited availability of burn surgery resources, including skin substitutes, at KCMC.

**Table 4:** Multivariable Logistic Regression for ICU Admission, In-Hospital Mortality, and Morbidity (Glasgow Outcome Score-Extended, GOS-E)

	**Admission to the ICU**				**In-hospital mortality**				**Poor recovery (GOS-E score 3-8)**			
**Characteristic**	**Odds Ratio**	**95% CI Lower**	**95% CI Upper**	***P*-value**	**Odds Ratio**	**95% CI Lower**	**95% CI Upper**	***P*-value**	**Odds Ratio**	**95% CI Lower**	**95% CI Upper**	***P*-value**
Sex, *N* (%)												
Male	0.341	0.098	1.191	.0919	0.597	0.186	1.914	.3858	0.681	0.300	1.545	.3577
Female	Ref				Ref							
Age, years	1.159	0.969	1.387	.1061	0.897	0.758	1.063	.2102	0.969	0.860	1.092	.6075
Where patient lives*, N* (%)												
Moshi urban	Ref				Ref							
Moshi rural	0.244	0.034	1.737	.1589	0.897	0.758	1.063	.1813	3.386	1.072	10.693	.0376
Other (rural surrounding areas)	0.626	0.157	2.498	.5069	1.986	0.487	8.102	.3391	1.734	0.680	4.423	.2493
Burn severity, per 10% change	2.580	1.786	3.727	<.0001	2.209	1.619	3.014	<.0001	1.537	1.236	1.912	.0001

### Time from burn injury to arrival at KCMC

**Figure 2:**
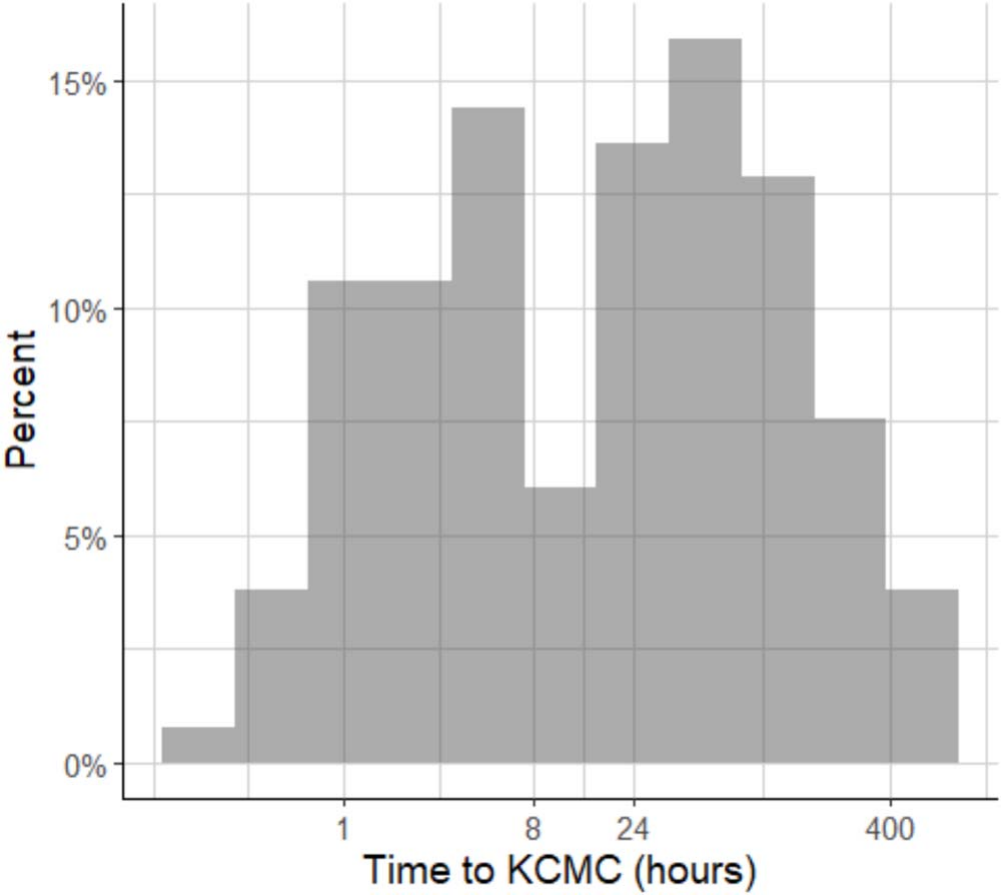
Histogram of Time from Burn Injury to Kilimanjaro Christian Medical Centre Arrival

The median time from burn injury to arrival at KCMC was 20.9 hours (range 3.1-76.1 hours), with 75% of patients spending time at an intermediary facility prior to arrival. Early resuscitation of burn-injured patient is critical, with cut-offs of 8 hours and 24 hours considered important markers for initiation of fluid resuscitation.^25^ In our cohort, 42.1% (*n* = 56) of patients arrived within 8 hours (considered *early*), while 48.1% (*n* = 64) arrived after 24 hours (considered *late*) (Figure 2). Time to arrival was not significantly associated with ICU admission, in-hospital mortality, or morbidity.

**Figure 3:**
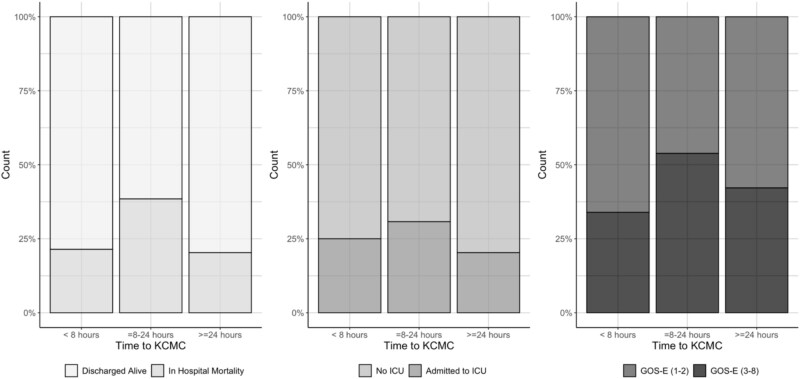
Bar Plot of Time from Burn Injury to KCMC Arrival by In-Hospital Mortality, ICU Admission, and Morbidity (GOS-E) with Colors Indicating the Proportion of Outcomes for Patients Arriving Within Each Time Frame. Abbreviation: GOS-E, Glasgow Outcome Score-Extended.

To further evaluate the bimodal distribution of time from burn injury to definitive burn care, we considered the 8-hour and 24-hour time markers from initial burn injury. There was no difference in in-hospital mortality between patients who arrived within 8 hours of their initial injury and those who arrived after 8 hours (21.4% vs 23.4%, *P* = .79). Similarly, there was no difference in in-hospital mortality between patients who arrived within 24 hours of their initial injury and those who arrived after 24 hours (24.6% vs 20.3%, *P* = .55). In Figure 3, which illustrates the distribution of in-hospital mortality, ICU admission, and functional morbidity stratified by time from burn injury to arrival at KCMC via bar plot, in-hospital mortality, ICU admission, and morbidity were proportionately highest among patients arriving between 8 and 24 hours.

Although the 8-24 hour group was the smallest (*n* = 13), a more detailed analysis demonstrated some possible patterns for this middle arrival group compared to those arriving *early* (<8 hours) and *late* (>24 hours) post-burn injury (Table 5**)**. Clinically, the 8-24 hour group had the highest proportion of severe burns (69.2%) compared to 32.1% in the early arrival group and 51.6% in the late arrival group (*P* = .002). On evaluation of pre-hospital characteristics, all of the patients in the 8-24 hour group were brought by ambulance from another facility (*P* = .001). None were initially treated at KCMC, in contrast to the early arrival group, where 46% presented directly to KCMC (*P* < .001).

## DISCUSSION

In this manuscript, we present a cohort of pediatric burn injury patients who arrived at tertiary-level burn care in Northern Tanzania and characterize the epidemiology, clinical presentation, and pre-hospital factors that impact their clinical outcomes. While there is growing data on pediatric injuries in LMIC settings, data specific to pediatric burn injuries continues to be quite limited.^9^ This study found that the mortality rate of our patient cohort was higher than in high-income country (HIC) settings but consistent with other LMIC settings, burn severity and abnormal vital signs on hospital arrival were associated with poor outcomes, and transport from intermediary facilities impacted survival in this patient population.

Our cohort had fairly even sex distribution, with most of the patients under 5 years of age. These demographics are similar to other descriptive studies of pediatric burn patients in both HIC and LMIC settings, including Tanzania.^26^^,^^27^ Scald was the primary mechanism of burn injury in our cohort, which is also consistent with other pediatric burn studies in LMIC settings globally.^28-32^

The in-hospital mortality rate of our pediatric burn cohort was 22.6%, which is notably higher than rates reported in HICs, but falls within the range observed across similar LMIC settings.^33^^,^^34^ In studies performed in the United States and Switzerland, pediatric burn mortality rates were less than 1%.^35-37^ Pediatric burn mortality rates reported in African institutions vary widely, from 3.2% at a tertiary-level burn unit in Addis Ababa, Ethiopia, to as high as 39.5% in a referral teaching hospital in Ibadan, Nigeria.^10^^,^^38-41^ In a cross-sectional analysis of 8 LMIC burn centers globally, including adult and pediatric patients, the mortality rate ranged from 5% to 42%.^42^ These variations highlight disparities in burn care resources and outcomes in pediatric patients across African LMICs that need to be addressed.

Clinical factors associated with poor outcomes in our patient cohort included burn severity and vital sign abnormalities. Burn severity independently predicted all 3 outcomes, and a TBSA threshold of approximately 25% marked an exponential increase in risk for poor outcomes. Patients presenting with extensive burns (20%-39% TBSA) had a better in-hospital mortality rate (21.9%) and morbidity (56.3% with good function at discharge) compared to patients with burns beyond 40% TBSA (in-hospital mortality rate of 67.9% with 25% having good function at discharge). These findings are consistent with other studies in pediatric and adult populations. Severity of burn injury by TBSA has been well described as a risk factor for poor outcomes, including mortality, in all settings.^43-45^ Our data also suggests that patients with 20%-39% TBSA have a decent chance of survival compared to patients with burns 40% TBSA or greater, with subspecialty burn care and resources currently available at KCMC, and may benefit from prioritized care.

**Table 5:** Characteristics of Burn Patients by Time from Burn Injury to KCMC Arrival

	**Overall**	**<8 hours**	**8-24 hours**	***>*24 hours**	
**Characteristic**	*n* = 133	*N* = 56	*N* = 13	*n* = 64	***P***
**Demographics**					
**Sex, *N* (%)**					.428
Male	65 (48.9%)	29 (51.8%)	8 (61.6%)	28 (43.8%)	
Female	68 (51.1%)	27 (48.2%)	5 (38.4%)	36 (56.2%)	
**Age, years, mean (SD)**	3.5 (3.4)	3.3 (3.2)	3.0 (3.0)	3.9 (3.7)	.493
**Age group, *N* (%)**					.3460^b^
1. Infant (0-1 year)	45 (33.8%)	20 (35.7%)	5 (38.5%)	20 (31.3%)	
2. Toddler (2-3 years)	40 (30.1%)	16 (28.6%)	4 (30.8%)	20 (31.3%)	
3. Preschool (4-5 years)	24 (18.0%)	13 (23.2%)	3 (23.1%)	8 (12.5%)	
4. Child (6-11 years)	17 (12.8%)	4 (7.1%)	0 (0.0%)	13 (20.3%)	
6. Teen (12-17 years)	7 (5.3%)	3 (5.4%)	1 (7.7%)	3 (4.7%)	
**Where patient lives, *N* (%)** **(**1 patient missing data)					<.001
Moshi urban	47 (35.6%)	31 (55.4%)	2 (16.7%)	14 (21.9%)	
Moshi rural	30 (22.7%)	13 (23.3%)	5 (41.7%)	12 (18.8%)	
Other (rural surrounding areas)	55 (41.7%)	12 (21.4%)	5 (41.7%)	38 (59.4%)	
**Patient lives with, *N* (%)** (1 patient missing data)					.112^b^
Single parent	25 (18.9%)	11 (19.6%)	3 (25.0%)	11 (17.2%)	
Both parents	94 (71.2%)	43 (76.8%)	9 (75.0%)	42 (65.6%)	
Grandparent/aunt/uncle	13 (9.8%)	2 (3.6%)	0 (0.0%)	11 (17.2%)	
**Number of children living in the home, *N* (%)** (1 patient missing data)					.237
1 (patient)	31 (23.5%)	13 (23.2%)	3 (25%)	15 (23.4%)	
2-3	75 (56.8%)	35 (62.5%)	8 (66.7%)	32 (50.0%)	
4-5	19 (14.4%)	8 (14.3%)	0 (0.0%)	11 (17.2%)	
6-7	6 (4.5%)	0 (0.0%)	1 (8.3%)	5 (7.8%)	
>7	1 (0.8%)	0 (0.0%)	0 (0.0%)	1 (1.6%)	
**Clinical characteristics**					
**Burn severity, *N* (%)**					<.001^b^
1. Mild burn (<10% BSA)	36 (27.1%)	24 (42.9%)	3 (23.1%)	9 (14.1%)	
2. Moderate burn (10%-19% BSA)	37 (27.8%)	14 (25.0%)	1 (7.7%)	22 (34.4%)	
3. Extensive burn (20%-39% BSA)	32 (24.1%)	4 (7.1%)	5 (38.5%)	23 (35.9%)	
4. Severe Burn (≥40% BSA)	28 (21.1%)	14 (25.0%)	4 (30.8%)	10 (15.6%)	
**Temperature, *N* (%)** (4 patients missing data)					.061^b^
Hypothermia (<36 °C)	8 (6.2%)	3 (5.6%)	2 (16.7%)	3 (4.8%)	
Normal (36-38 °C)	107 (82.9%)	49 (90.7%)	8 (66.7%)	50 (79.4%)	
Fever (>38 °C)	14 (10.9%)	2 (3.7%)	2 (16.7%)	10 (15.9%)	
**Tachypnea, *N* (%)**					.256^b^
No	118 (88.7%)	51 (91.1%)	13 (100.0%)	54 (84.4%)	
Yes	15 (11.3%)	5 (8.9%)	0 (0.0%)	10 (15.6%)	
**Hypotension, *N* (%)** (24 patients missing data)					.103^b^
No	105 (96.3%)	43 (91.5%)	10 (100.0%)	52 (100.0%)	
Yes	4 (3.7%)	4 (8.5%)	0 (0.0%)	0 (0.0%)	
**Hypoxemia, *N* (%)** (2 patients missing data)					.211^b^
No	122 (93.1%)	53 (96.4%)	11 (84.6%)	58 (92.1%)	
Yes	9 (6.9%)	2 (3.6%)	2 (15.4%)	5 (7.9%)	
**Tachycardia, *N* (%)**					.558^b^
No	118 (88.7%)	49 (87.5%)	13 (100.0%)	56 (87.5%)	
Yes	15 (11.3%)	7 (12.5%)	0 (0.0%)	8 (12.5%)	
**Required intubation in the ED, *N* (%)**					.200^b^
No	121 (91.0%)	48 (85.7%)	12 (92.3%)	61 (95.3%)	
Yes	12 (9.0%)	8 (14.3%)	1 (7.7%)	3 (4.7%)	
**Patients meeting SIRS criteria** ^a^ **upon ED arrival, *N* (%)**					.786^b^
No	124 (93.2%)	53 (94.6%)	12 (92.3%)	59 (92.2%)	
Yes	9 (6.8%)	3 (5.4%)	1 (7.7%)	5 (7.8%)	
**Patients meeting SIRS criteria** ^a^ **+ hypotension upon ED arrival, *N* (%)**					NA^c^
No	132 (99.2%)	55 (98.2%)	13 (100.0%)	64 (100.0%)	
Yes	1 (0.8%)	1 (1.8%)	0 (0.0%)	0 (0.0%)	
**Pre-hospital characteristics**					
**Mechanism of transport to KCMC, *N* (%)**					<.001^b^
Ambulance from other hospital	87 (65.4%)	24 (42.9%)	13 (100.0%)	50 (78.1%)	
Private car	27 (20.3%)	17 (30.4%)	0 (0.0%)	10 (15.6%)	
Hired transportation	16 (12.0%)	12 (21.4%)	0 (0.0%)	4 (6.3%)	
Other	3 (2.3%)	3 (5.4%)	0 (0.0%)	0 (0.0%)	
**First health facility treated at, *N* (%)** (33 patients missing data)					<.001^b^
KCMC	25 (25.0%)	23 (46.0%)	0 (0.0%)	2 (4.8%)	
Dispensary/health center	25 (25.0%)	8 (16.0%)	3 (37.5%)	14 (33.3%)	
District/regional hospital	50 (50.0%)	19 (38.0%)	5 (62.5%)	26 (61.9%)	

^a^SIRS criteria: *>*2 of the following (1 of which must be a normal temperature): tachycardia, tachypnea (or mechanical ventilation), temperature abnormality; laboratory data not available in registry. Abbreviation: KCMC, Kilimanjaro Christian Medical Centre. *P* values assessed using chi-square test of association (categorical) or analysis of variance/*t*-test (continuous), unless otherwise noted.

^b^ *P* value assessed using Fisher’s exact test.

^c^Not enough events for valid statistical inference

Previous studies of general injury patients and burn injury patients in the pediatric population have found vital sign abnormalities to be associated with poor outcomes. A study of pediatric injury patients at KCMC found that patients with tachycardia, hypoxia, hypotension, and abnormal GCS on arrival had an increased risk of mortality.^12^ A study in Johannesburg, South Africa, found that hypotension in burn patients of all ages within 48 hours of arrival to a specialized burn center was associated with intensive care requirement (mechanical ventilation, inotropes, and dialysis) during admission and significantly higher odds of mortality.^46^ Additionally, organ failure has been found to contribute significantly to morbidity and mortality in pediatric patients with burn injury.^47^^,^^48^ These studies suggest that vital signs and lab markers of end-organ dysfunction are critical for monitoring these young patients. The pediatric injury registry at KCMC was not established as a burn-specific database and, unfortunately, does not collect laboratory data at this time. The WHO Global Burn Registry has been adapted for low-resource settings but is still limited in its ability to collect laboratory data due to diagnostic availability and associated costs in low-resource areas.^49^ As pediatric injury registries develop further, it will be imperative to ensure burn-specific data is included – including accurate TBSA, depth of burn, and presence of inhalational injury^50^—and consider the potential for low-cost and point-of-care diagnostics in identifying end-organ involvement.^51^ Our data supports previous study findings that identifying high-risk pediatric burn patients early, via TBSA and other physiologic markers, is essential for triage and resource allocation as well as development of clinical care guidelines applicable to low-resource settings like Northern Tanzania.

Pre-hospital factors associated with ICU admission and in-hospital mortality in our cohort included ambulance transport and regional or district hospitals as the initial site of care. Additionally, while time from initial burn injury to definitive burn care at KCMC did not show a simple linear relationship with study outcomes, it revealed a sub-group of patients arriving at tertiary burn care 8-24 hours after injury that requires further study. Previous publications have shown that children with burn injuries from rural settings all over the globe experience delays in receiving medical treatment due to many barriers, including geography.^32^^,^^52^^,^^53^ Delays in injury care in LMIC settings are associated with worse outcomes.^54^ Ambulance and pre-hospital infrastructure is limited in the majority of LMIC settings and has been linked to overall lack of emergency care services and worse outcomes for pediatric and adult patients.^55-57^ Access to early resuscitative care in burns is critical, thus it would be expected to see a linear relationship showing better outcomes in patients arriving early to burn care compared to those arriving later in our study cohort.^25^ The 8-24-hour sub-group had worse clinical outcomes instead, despite not being the most delayed arrival group. These findings could suggest those arriving to tertiary care after 24 hours had less severe injuries and were able to survive the critical first 24 hours post-burn with limited resuscitation.^25^ Our findings suggest delays to prompt pediatric burn care exist in Northern Tanzania, and strengthening of pre-hospital systems is needed to expedite treatment and improve clinical outcomes. Further study with a larger number of patients arriving 8-24 hours post-burn injury is also needed to better understand how this mid-delay group of patients differs from other sub-groups to target interventions focused on improving morbidity and mortality.

## LIMITATIONS

This study included multiple limitations. This study was facility-based at a tertiary level zonal referral hospital with pediatric subspecialty services, including burn care. The injury registry does not include data regarding pre-hospital treatment received at other medical facilities, nor post-discharge follow-up data. There is potential for survivorship sampling bias in our results, and we are missing important community-level data regarding care of patients prior to reaching KCMC. Our setting and sample miss patients with critical burns that resulted in death prior to KCMC arrival, and patients not critical enough to warrant transfer from intermediary facilities. The registry used for data collection was created to capture all pediatric injury patients and is not burn-specific. We based severity of burn injury in our patients on TBSA due to variability in reporting burn injury depth in medical records. Our current analysis does not include all variables of standardized burn-specific registries, like inhalation injuries, depth of burn injury, or laboratory values that can identify patients with end-organ damage. Some patients in our cohort had missing data variables, as described in Tables 3 and 5. There is also potential for data error as research assistants obtained variables, including vital signs, from the KCMC medical record system. The research assistants were trained heavily in this step of clinical documentation, but could contribute to errors. Finally, descriptive epidemiologic studies are limited by their lack of hypothesis testing. In resource-limited settings, however, they are essential for documenting surgical care and guiding future research. Peer-reviewed dissemination remains crucial for advancing global surgical equity.

## CONCLUSION

The mortality rate of our pediatric patients accessing tertiary burn care in Northern Tanzania was consistent with similar African LMIC settings, and severity of burn injury was independently associated with ICU admission, in-hospital mortality, and morbidity. This study highlights the urgent need to address gaps in burn injury prevention, community education on timely burn care, and pre-hospital and inter-facility referral systems for pediatric burn patients in Northern Tanzania. Improving early access to resuscitation and definitive care, particularly for children with severe burn injuries, is vital to reducing morbidity and mortality in this young population.

## Notes

Author contributions: Kajsa Vlasic (Conceptualization [lead], Data curation [equal], Formal analysis [equal], Funding acquisition [lead], Investigation [lead], Methodology [lead], Project administration [lead], Resources [lead], Supervision [lead], Validation [lead], Visualization [lead], Writing—original draft [lead], Writing—review & editing [lead]), Theresia Mwakyembe (Conceptualization [supporting], Investigation [supporting], Supervision [supporting], Visualization [supporting], Writing—review & editing [supporting]), Francis Sakita (Conceptualization [supporting], Investigation [supporting], Supervision [supporting], Visualization [supporting], Writing—review & editing [supporting]), Irma Fleming (Supervision [supporting], Visualization [supporting], Writing—review & editing [supporting]), Giavonni Lewis (Supervision [supporting], Visualization [supporting], Writing—review & editing [supporting]), Getrude Nkini (Data curation [lead], Investigation [equal], Project administration [equal], Resources [equal], Writing—review & editing [supporting]), Nancy Mmary (Data curation [equal], Project administration [supporting], Resources [supporting], Writing—review & editing [supporting]), Deus Marandu (Data curation [supporting], Investigation [supporting], Writing—review & editing [supporting]), Nora Fino (Conceptualization [supporting], Formal analysis [lead], Methodology [equal], Resources [supporting], Software [supporting], Writing—review & editing [supporting]), Jonah Holiday (Investigation [supporting], Writing—review & editing [supporting]), Melissa H. Watt (Conceptualization [supporting], Funding acquisition [supporting], Supervision [supporting], Writing—review & editing [supporting]), Catherine A. Staton (Conceptualization [supporting], Formal analysis [supporting], Visualization [supporting], Writing—review & editing [supporting]), Elizabeth M. Keating (Conceptualization [supporting], Data curation [lead], Formal analysis [supporting], Funding acquisition [supporting], Investigation [supporting], Methodology [supporting], Project administration [lead], Resources [lead], Supervision [supporting], Visualization [supporting], Writing—review & editing [supporting]), and Blandina T. Mmbaga (Conceptualization [supporting], Data curation [supporting], Project administration [supporting], Resources [supporting], Supervision [supporting], Visualization [supporting], Writing—review & editing [supporting]).

Funding: This study was supported by a pilot grant to K.V. from the University of Utah Department of Pediatrics Health Equity Research Core, with funding from the Intermountain Foundation at Primary Children’s Hospital. E.M.K. is supported in this work by the Eunice Kennedy Shriver National Institute of Child Health and Human Development (grant K23 HD112548). The content is solely the responsibility of the authors and does not necessarily represent the official views of the University of Utah, the Intermountain Foundation at Primary Children’s Hospital, or the National Institutes of Health. The funders had no role in the decision to publish or preparation of the manuscript. There was no additional external funding received for this study.
